# Association of Nepeta cataria L. essential oil and eugenol: synergism and safe anesthesia in tambaqui Colossoma macropomum (Cuvier, 1818)

**DOI:** 10.1007/s11259-026-11298-x

**Published:** 2026-05-28

**Authors:** Taissa Viana Damasceno, Thaysa de Sousa Reis, Axell Lins, Priscille Fidelis Hartcopff, Marcelo Victor dos Santos Brito, Daniella Rocha Bittencourt, Luiz Fernando Duarte de Andrade Junior, Alicia Chaves Manito, Gabriela de Paula Goyana, Cibely Camille Fernandes Ferreira, Dayanne Raquel de Sousa Barros, Luana Vasconcelos de Souza, Nilton Akio Muto, Moisés Hamoy

**Affiliations:** 1 Laboratory of Pharmacology and Toxicology of Natural Products, Institute of Biological Sciences, Federal University of Pará (ICB/UFPA), Belém, Pará Brasil; 2 Center for Valorization of Amazonian Bioactive Compounds- Instituto de Ciências Biológicas, Federal University of Pará, Belém, Pará Brasil; 3 Federal University of Pará, R. Augusto Corrêa, 01, Guamá, Belém, 66075-110 Pará Brazil

**Keywords:** Adjuvant, Pharmaceutical, Anesthesia, *Colossoma macropomum*, Synergism

## Abstract

Anesthetics are widely employed in aquaculture and in research environments utilizing fish as experimental models, with the aim of mitigating stress and hypermotility during handling procedures. Among the most commonly used anesthetic agents, eugenol stands out for its suitability across multiple species, enabling safer handling and transportation while minimizing physiological impact on the animals. For the experimental procedure, three oil mixtures were prepared in a 1:1 ratio. The first contained 50% eugenol and 50% Nepeta cataria essential oil (ENCO); the second mixture was 50% eugenol and 50% corn oil (*Zea mays*) (ECO); and the third was 50% Nepeta cataria essential oil and 50% corn oil (NCCO). The present study proposes a behavioral and electrophysiological assessment of *Colossoma macropomum* subjected to immersion baths containing different concentrations of *Nepeta cataria* L. 50% and eugenol 50%, in addition to investigating the underlying components of the synergism and the mechanism of action of eugenol. A total of 288 juvenile specimens, of mixed sex, with a mean body mass of 25.34 ± 3.2 g, were used. Experiment 1: assessed the synergistic potentiation between the compounds, based on latency times for anesthetic induction and recovery. Experiment 2: performed electrophysiological analyses of muscle (EMG) and cardiac (ECG) functions. Experiment 3: evaluated the influence of flumazenil on the activation of the anesthetic agents. Evaluations were conducted during 10-minute immersion baths at concentrations of 30 µL·L⁻¹, 35 µL·L⁻¹, 40 µL·L⁻¹, 45 µL·L⁻¹, and 50 µL·L⁻¹. The combination of Nepeta cataria essential oil and eugenol promotes good muscle relaxation and minor alterations in the cardiovascular system, promoting easily reversible bradycardia. The findings indicate that the combination of *Nepeta cataria* essential oil and eugenol constitutes a promising and safe alternative, due to the synergistic reduction in induction time and the maintenance of cardiac safety, which allowed for a reduction in the concentration required without compromising the maintenance of the anesthetic plane. Furthermore, flumazenil was found to exert a significant influence on both the induction process and the recovery phase of anesthesia.

**Supplementary Information:**

The online version contains supplementary material available at 10.1007/s11259-026-11298-x.

## Introduction

*Colossoma macropomum* (tambaqui), the most widely farmed fish in South America, is native to the Amazon Basin (Pires et al. 2018) and ranks as the second most produced aquatic organism in Brazil, surpassed only by Nile tilapia (*Oreochromis niloticus*) (Peixe 2023). In 2022, its production exceeded 109,000 tons, underscoring its economic importance for the region (Peixe 2023). Given its significant zootechnical value, routine management procedures must be conducted safely to prevent stress and reduce animal mortality (GIMBO et al., 2008; Martos-Sitcha et al. 2020). Accordingly, the use of anesthetic or sedative substances is essential to minimize animal losses during handling, prevent injuries, and facilitate various procedures (Aydın and Barbas 2020; da Paz et al. 2024; de Sousa Reis et al. 2024).

The use of anesthetics helps reduce physical damage caused by injuries during handling and transportation, thereby decreasing the susceptibility of fish to pathogens and infectious diseases (Sloman et al. 2019; Vergneau-Grosset and Cruz Benedetti 2022). In light of this, anesthesia protocols are routinely established to ensure a smooth transition during induction and recovery phases, aiming to achieve the desired anesthetic plane (Aydın and Barbas 2020; Fujimoto et al. 2018). However, the use of an inadequate anesthetic protocol may compromise not only fish welfare but also lead to unsatisfactory outcomes in aquaculture practices (Martins et al. 2019). Therefore, investigating safe and effective anesthetic agents is essential to address this growing demand for animal welfare.

According to Ross & Ross (2009), the ideal short-duration anesthesia in fish should have a rapid and safe induction, with 2.5 min for induction and recovery. However, according to Zahl et al. 2012; all anesthesia intended for surgical procedures should include the use of analgesics to avoid discomfort from pain.

Several synthetic and natural anesthetic agents have been studied to assess their safety and efficacy in fish. Among natural anesthetics, clove oil has been widely used to immobilize and suppress the sensory systems of fish during invasive procedures. It has proven particularly suitable for use in commercial aquaculture settings due to the reversibility of its effects and its anesthetic safety profile (Aydın and Barbas 2020; Javahery et al. 2012). Studies indicate that eugenol exerts its effects through mechanisms such as sodium channel blockade and increased GABAA receptor activity, acting as an agonist (Aoshima and Hamamoto 1999; Cho et al. 2008; Ding et al. 2014; Kheawfu et al. 2022). Furthermore, the potential of other essential oils, such as those derived from *Lippia alba* (de Freitas Souza et al. 2018) and *Nepeta cataria* (dos Santos et al. 2024), has also shown promising anesthetic and sedative effects in fish. According to Dos Santos et al. (2024), it was possible to evaluate the reversibility of cardiac and respiratory effects in *Colossoma macropomum* using reduced concentrations of *Nepeta cataria* essential oil. Thus, various essential oils have been investigated to validate their potential anesthetic effects.

The synergism between substances as a strategy to promote more effective anesthetic effects is also discussed in the literature. When two or more drugs are combined and elicit a response greater than expected, it is considered that a synergistic effect occurs between them (Tchobanov et al. 2024). Accordingly, several studies emphasize the investigation of alternative protocols that enable synergism to enhance the efficacy and safety of anesthesia in fish (Brandão et al. 2021). This is because the combination of drugs with distinct mechanisms of action can potentiate their effects (Tchobanov et al. 2024). The study conducted by Tchobanov et al. (2024) demonstrated a reduction in induction and recovery times in *Sparus aurata* when lidocaine was combined with clove oil or 2-phenoxyethanol. Similarly, the research by (Nuanmanee et al. 2024) showed increased anesthetic efficiency in guppies (*Poecilia reticulata*) when eugenol was combined with 1,8-cineole. These findings underscore the importance of studies focused on substance synergism, highlighting its crucial role in ensuring safe and effective anesthesia.

This study aims to evaluate the anesthesia induced by the combination of eugenol and Nepeta cataria essential oil in *Colossoma macropomum*, considering behavioral and electrophysiological parameters during anesthetic induction and recovery. Additionally, we investigated the contribution of GABA benzodiazepine receptor activation to anesthesia using flumazenil.

## Materials and methods

### Experimental animals and acclimatization period

This study was approved by the Animal Experimentation Ethics Committee of the Federal University of Pará (CEUA - UFPA − 3898260521).The individuals used in this study consisted of a total of 288 juvenile *Colossoma macropomum* (25.34 ± 3.2 g). The animals were acclimatized in 5 aquariums of 250 L with artificial aeration, with approximately 60 animals per aquarium. The acclimatization period lasted 20 days at the Experimental Bioterium of the Pharmacology and Toxicology of Natural Products Laboratory at the ICB - Federal University of Pará (UFPA), with controlled temperature (25 °C to 28 °C) and a 12-hour light: 12-hour dark photoperiod. They were fed commercial feed (32% protein) ad libitum. Water quality parameters, including temperature (°C), pH, and dissolved oxygen (DO), were monitored and maintained using a multiparametric measuring device (AK-88). It is worth noting that this methodology has been widely used in our laboratory and has served as a basis for other works by our group, such as (Barbas et al. 2017; dos Santos et al. 2024). The animals were fasted for 6 h prior to the anesthetic procedure to prevent regurgitation.

### Drug acquisition and experimental design

The substances used in this study were the essential oil of *Nepeta cataria*, acquired from the Lazslo laboratory (CNPJ: 37.831.582/0001–68) (Beleza do Campo ™, Barretos, SP). The composition of the essential oil was as follows: 0.6% β-pinene, 0.2% piperitone, 77.6% nepetalactone, 7.6% dihydronepetalactone, 3.2% β-caryophyllene, 1.7% α-humulene, 1.1% farnesene, and 0.6% caryophyllene oxide, according to the supplier’s information. Additionally, pure eugenol, in a 20 mL vial, was acquired from Maquira (Av. Champagnat, 1910, Franca – SP, Brazil). Refined corn oil, 250 mL, was obtained from the company Mundo dos Óleos LTDA, based in Brasília-DF, Brazil.

For the experimental procedure, three oil mixtures were prepared in a 1:1 ratio. The first contained 50% eugenol and 50% *Nepeta cataria* essential oil (ENCO); the second mixture was 50% eugenol and 50% corn oil (*Zea mays*) (ECO); and the third was 50% *Nepeta cataria* essential oil and 50% corn oil (NCCO).

Due to their low solubility in water, stock solutions made with the oil mixtures (ENCO, ECO, and NCCO) were prepared and pre-diluted in ethanol (70%) in a 1:9 ratio, from which aliquots were taken for the anesthesia test. Both products were stored in amber glass bottles at 4 °C until use.

After the acclimatization period, the animals were randomly assigned to the following treatment groups: (a) control (baseline recording), (b) vehicle-treated (70% ethanol at 1 mL/L of water), (c) 30 µL.L^− 1^, (d) 35 µL.L^− 1^, (e) 40 µL.L^− 1^, (f) 45 µL.L^− 1^, and (g) 50 µL.L^− 1^ (total volume of the oil mixtures). The concentrations were applied to fish submerged in the oil mixtures (ENCO, ECO, and NCCO), followed by recovery. Each treatment lasted 10 min, and a sample size of *n* = 9 per treatment was used for each recording. The latency phase was assessed, and cardiac recordings were taken for analysis. After the induction of anesthesia, the animals were transferred to anesthetic-free water, and recovery was monitored for 10 min. The experimental aquariums contained a water volume of 5 L; after each experiment, the aquarium was cleaned and the water was renewed.

The concentrations of *Nepeta cataria* essential oil were previously evaluated by dos Santos et al. 2024.

### Experiment 1: behavioral analysis

Considering the exposure time to ENCO, ECO, and NCCO concentrations of 30 µL.L^− 1^, 35 µL.L^− 1^, 40 µL.L^− 1^, 45 µL.L^− 1^, and 50 µL.L^− 1^ in immersion baths, the latency to the loss of posture reflex behavior was evaluated. This is characterized by the maintenance of lateral decubitus during the experiment. After a 10-minute period, the animals were removed from the oil mixture treatment and placed in aquarium water without anesthetics to assess the latency to recover the posture reflex, which was characterized by maintenance during recovery (Hamoy et al. 2023; Vieira et al. 2023). To avoid bias in the research, the observer of the behavior was unaware of the concentration and combination of anesthetics being used.

### Experiment 2: electrocardiogram (ECG) and electromyogram (EMG) analysis

For ECG recording, electrodes were made of silver wire (925), with a diameter of 0.3 mm and a length of 10 mm and immersed for 24 h in sodium hypochlorite. The reference electrode was positioned according to the cardiac vector, with the negative pole at the base of the heart and the positive pole at the apex of the heart, positioned in the ventral portion of the left opercular opening, 0.2 mm before the end of the opercular cavity. The recording electrode was inserted 2.0 mm from the right opercular opening. They were then connected to a high-impedance amplifier (Grass Technologies, Model P511). The amplifier was tuned with a 0.3 ohm high-pass filter and a 300 Hz low-pass filter. The recordings were used to analyze heart rate (bpm), QRS complex amplitude (mV), QRS complex duration (ms), R-R intervals (ms), P-Q intervals (ms), and S-T intervals (ms) (da Paz et al. 2024).

Muscle activity (dorsal muscle) was recorded during induction and recovery from anesthesia. The electrodes were made of 925 silver with a diameter of 0.5 mm and a length of 15 mm. The electrodes were made in a conjugated manner with a spacing of 5 mm and insulated with liquid insulation. In the EMG recordings, the powers during muscle contraction in animals subjected to the immersion bath with ENCO and during anesthetic recovery (mV²/Hz) were evaluated. Each recording lasted 10 min. Data recording and analysis was done according to Hamoy et al. (2023). During the electrode insertion procedure, the fish were gently removed from the aquarium and wrapped in a damp cloth. Subsequently, cotton impregnated with 2% lidocaine was used at the electrode insertion site. After the electrodes were attached, the animals were placed in a smaller aquarium inside the Faradey cage, where they remained for 10 min before the recording began.

### Experiment 3: Test with flumazenil to demonstrate synergistic activity

For this stage of the project, 1 mg/kg i.p. of flumazenil (Yanqing Cao and Yu 2019) was used 30 min before anesthetic induction with ECO and ENCO at concentrations of 30 µL.L-1, 35 µL.L-1, 40 µL.L-1, 45 µL.L-1 and 50 µL.L-1 and a vehicle group to evaluate the delay in induction and recovery from anesthesia and the possible interference of flumazenil (Lanexat^®^) 0.1 mg/mL. The vehicle group did not show loss of postural reflex and flumazenil did not cause effects related to the analysis in the observation time; therefore, these data were not shown in the study.

### Statistical analysis

For the evaluation of the behavioral experiment, the non-parametric Student’s t-test was used, and the specific U test was performed using the Python program with the TestCase library (unittest). After verifying compliance with the assumptions of normality and homogeneity of variance through the *Kolmogorov-Smirnov and Levene tests*,* performed using the Python program with the scipy.stats library respectively*, comparisons of mean power values were performed using a two-way ANOVA (concentration and induction and recovery times factors), followed by Tukey’s test. The GraphPad Prism^®^ 8 software was used for the analyses, and p-values < 0.05, < 0.01, and < 0.001 were considered statistically significant in all cases.

## Results

### Behavioral analysis

Behavioral data indicated a loss of posture reflex in the ENCO and ECO oil mixtures; however, no loss of posture reflex was observed for the NCCO treatment in the fish. Behavioral analysis showed that, at all concentrations, the ECO treatment (eugenol + corn oil) resulted in significantly longer latency times for the loss of posture reflex compared to ENCO (eugenol + *Nepeta cataria*). This suggests that the essential oil of *Nepeta cataria* combined with eugenol was more effective and rapid in reducing the time required for the fish to lose the posture reflex compared to the combination of eugenol with corn oil. The difference between treatments is statistically significant at all analyzed concentrations, as indicated by the non-parametric T-test with *p* < 0.001. For the ENCO treatment, there was a reduction in latency for the loss of posture reflex in the following groups: 30 µL. L^− 1^ (36.59%); 35 µL. L^− 1^ (46.81%); 40 µL. L^− 1^ (33.65%); 45 µL. L^− 1^ (32.86%); 50 µL. L^− 1^ (49.68%), presented in Table 1.

**Table 1:** Latency in seconds (s) for the loss of posture reflex behavior for the combination of 50% eugenol with 50% Nepeta cataria essential oil (ENCO) compared to 50% eugenol with 50% corn oil (Zea mays) (ECO). T-test (non-parametric test) *p* < 0.001

Treatment/Oils	30µL. L^− 1^	35µL. L^− 1^	40µL. L^− 1^	45µL. L^− 1^	50µL. L^− 1^
ENCO	216.6 ± 11.91	149.3 ± 9.38	138.0 ± 7.69	96.0 ± 7.81	63.7 ± 7.04
ECO	341.6 ± 31.68 ***	280.7 ± 26.30***	208.3 ± 11.36***	143.3 ± 6.10***	126.6 ± 10.25***

The results presented in Table 2, indicating the recovery period for the posture reflex, showed that at lower concentrations (30 µL. L^− 1^ and 35 µL. L^− 1^), there was no significant difference between the ENCO and ECO treatments, suggesting that at these lower concentrations, both treatments have similar effects on the recovery of the posture reflex. However, the groups treated with ENCO exhibited longer latency in the groups treated with 40 µL. L^− 1^ (30.15%); 45 µL. L^− 1^ (22.28%); and 50 µL. L^− 1^ (16.43%).

**Table 2:** Latency in seconds (s) for the recovery of the posture reflex for the combination of 50% eugenol with 50% Nepeta cataria essential oil (ENCO) compared to 50% eugenol with 50% corn oil (Zea mays) (ECO). T-test (non-parametric test) *p* < 0.001

Treatment/Oils	30µL. L^− 1^	35µL. L^− 1^	40µL. L^− 1^	45µL. L^− 1^	50µL. L^− 1^
ENCO	178.9 ± 12.19	190.0 ± 7.21	238.8 ± 16.32	247.8 ± 24.24	269.0 ± 20.96
ECO	172.0 ± 28.20	165.7 ± 20.32	166.8 ± 14.47***	196.1 ± 5.98 ***	224.8 ± 11.23***

### Electromyographic recordings indicate that ENCO induced a loss of muscle tone during treatment and demonstrated reversibility during recovery

**Fig. 1:**
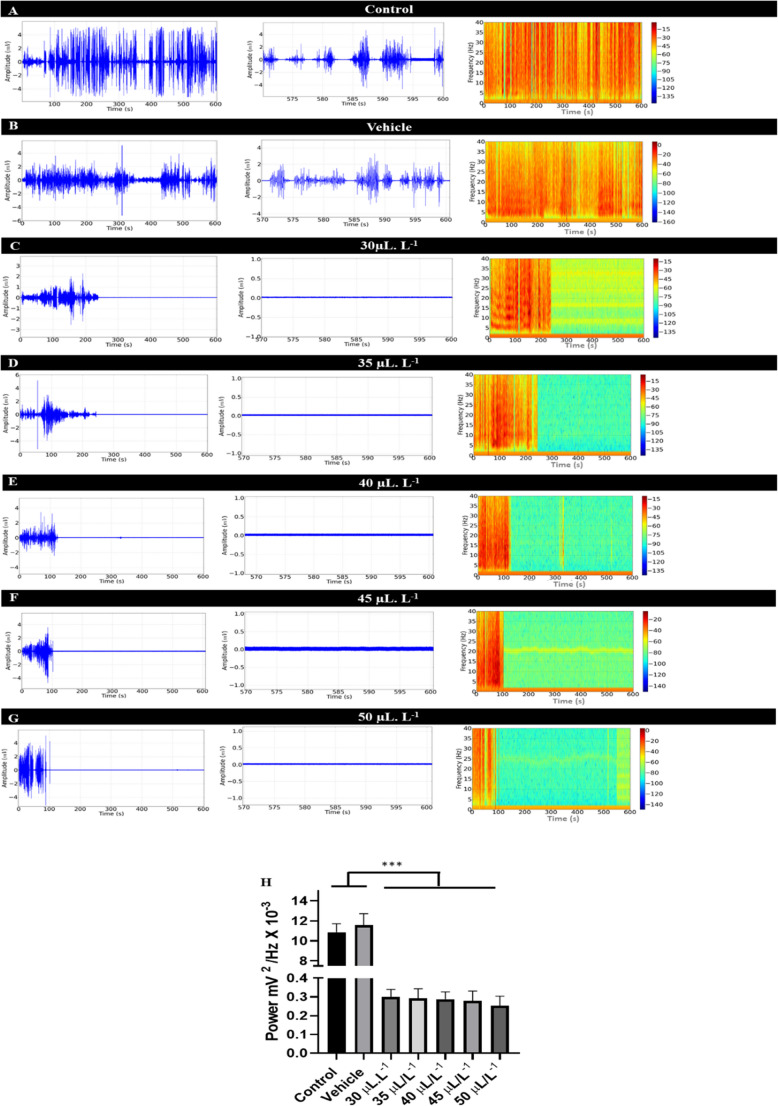
Demonstration of dorsal muscle activity recorded by the electrode. Electromyographic (EMG) trace with a duration of 600 s of muscle activity during swimming (Left), amplification of the final 30 s of the EMG records (570–600 s) showing the trace pattern (Center), and spectrogram showing the power distribution of EMG records across frequencies up to 40 Hz (Right), for the following groups: Control (**A**); Vehicle (**B**); treated with 30 µL.L^− 1^ (**C**); treated with 35 µL.L^− 1^ (**D**); treated with 40 µL.L^− 1^ (**E**); 45 µL.L^− 1^ (**F**); and 50 µL.L^− 1^ (**G**). The graph shows the power of muscle contractions on a linear scale during the final 30 s of immersion bath recording with 30 µL.L^− 1^, 35 µL.L^− 1^, 40 µL.L^− 1^, 45 µL.L^− 1^, and 50 µL.L^− 1^ ENCO (**H**). (ANOVA followed by Tukey *p < 0.05, **p < 0.01, ***p < 0.001, *n* = 9)

Activity of the dorsal muscle in tambaqui (*Colossoma macropomum*) during swimming for the control group showed an amplitude of 4 mV (Fig. 1A, left). The amplified recording illustrates the characteristics of intense muscle contraction over a 30-second period (Fig. 1A, center), with energy distribution during contraction in the 1 to 40 Hz frequency range, where higher power is observed according to the intensity of muscle contraction (reddish coloration in the spectrogram) (Fig. 1A, right). Figure 1B shows the electromyographic (EMG) recording for the vehicle group, which is similar to the control group.

During the 10-minute immersion bath treatment with 30 µL. L^− 1^ ENCO, a gradual decrease in muscle contraction was observed. However, analysis of the last 30 s of the recording revealed complete muscle relaxation, which is evident in the trace pattern showing silence in muscle activity. This result was also observed for the treatments with 35 µL. L^− 1^, 40 µL. L^− 1^, 45 µL. L^− 1^, and 50 µL. L^− 1^, represented by Figs. 1D, E, F, and G. Thus, the greater the contraction induced by the treatment, the more rapidly muscle relaxation occurs.

The mean power of muscle contractions during contact with ENCO at different concentrations was evaluated in Fig. 1H. Normal muscle contraction in the control group exhibited a mean of 10.86 ± 0.85 mV²/Hz × 10⁻³, which was similar to the vehicle group (*p* = 0.0848), but higher than the treated groups. The treated groups showed similar muscle relaxation (*p* = 0.999).

**Fig. 2:**
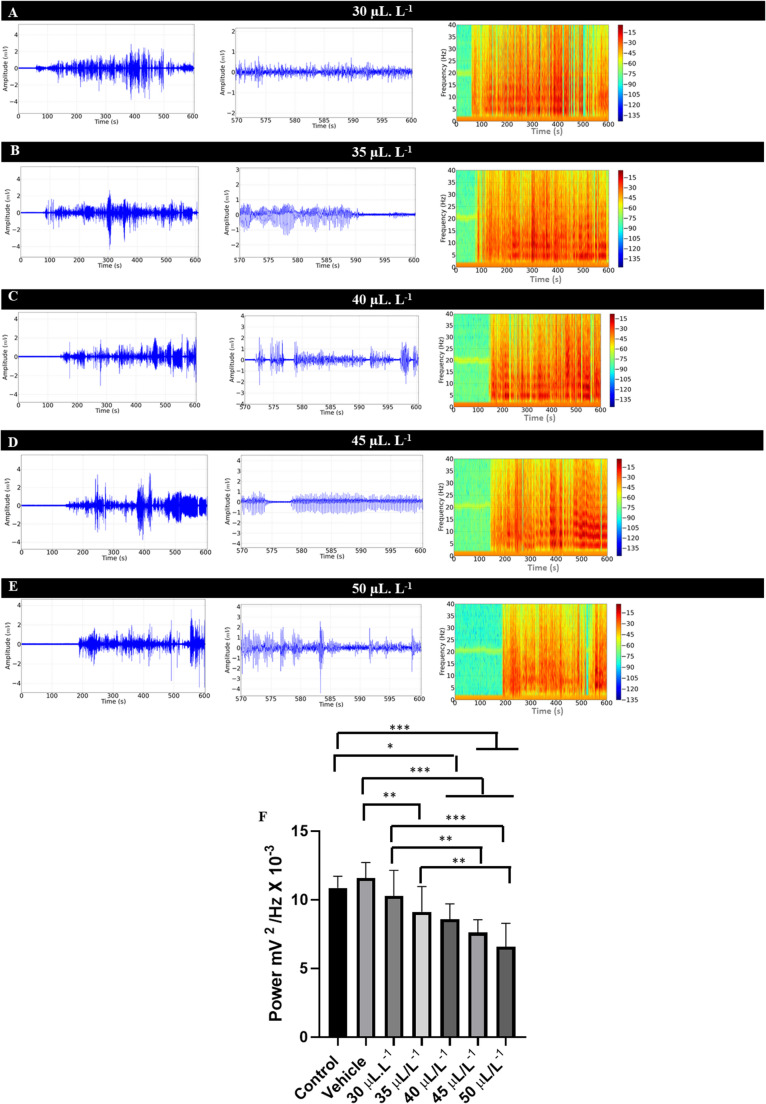
Dorsal muscle activity during recovery after immersion bath in groups treated with ENCO (left), amplification of muscle activity during the final 30 s of recording (570–600 s) (center), and spectrogram obtained from the electromyographic trace during 10 min of recovery (right), for the following groups: treated with 30 µL.L^− 1^ (**A**); treated with 35 µL.L^− 1^ (**B**); treated with 40 µL.L^− 1^ (**C**); 45 µL.L^− 1^ (**D**); and 50 µL.L^− 1^ (**E**). The graph shows the power of muscle contractions on a linear scale during the final 30 s of the recording during recovery from immersion bath with 30 µL.L^− 1^, 35 µL.L^− 1^, 40 µL.L^− 1^, 45 µL.L^− 1^, and 50 µL.L^− 1^ ENCO (**F**). (ANOVA followed by Tukey *p < 0.05, **p < 0.01, ***p < 0.001, *n* = 9)

To assess the recovery of muscle activity, recordings were made after immersion baths at different concentrations of ENCO in aquarium water. This allowed for the evaluation of muscle activation intensity. For the groups treated with 30 µL. L^− 1^, 35 µL. L^− 1^, 40 µL. L^− 1^, 45 µL. L^− 1^, and 50 µL. L^− 1^, muscle activity revealed the reversibility of the treatment, with latency for muscle recovery varying according to concentration. Higher concentrations corresponded to longer latency periods for muscle recovery (Figs. 2A, B, C, D, E and F). The spectrograms show an increase in recorded muscle activity indicated by the potential difference between the reference and recording electrodes (Figs. 2A, B, C, D, and E right).

The linear mean power analysis for muscle contraction during recovery in the control group was 10.86 ± 0.85 mV²/Hz × 10⁻³, which was similar to the vehicle group and those treated with 30 µL. L^− 1^ and 35 µL. L^− 1^ (*p* = 0.1417). The group treated with 30 µL. L^− 1^ (10.29 ± 1.85 mV²/Hz × 10⁻³) was similar to the group treated with 40 µL. L^− 1^(*p* = 0.1625). The group treated with 35 µL. L^− 1^ (9.12 ± 1.84 mV²/Hz × 10⁻³) was similar to the groups treated with 40 µL. L^− 1^ and 45 µL. L^− 1^ (*p* = 0.2743). The group treated with 40 µL. L^− 1^ (8.59 ± 1.11 mV²/Hz × 10⁻³) was similar to the groups treated with 45 µL. L^− 1^ and 50 µL. L^− 1^ (*p* = 0.0563) (Fig. 2F).

### Treatment with ENCO concentrations demonstrated safety in maintaining cardiac function during anesthetic induction and recovery

**Fig. 3:**
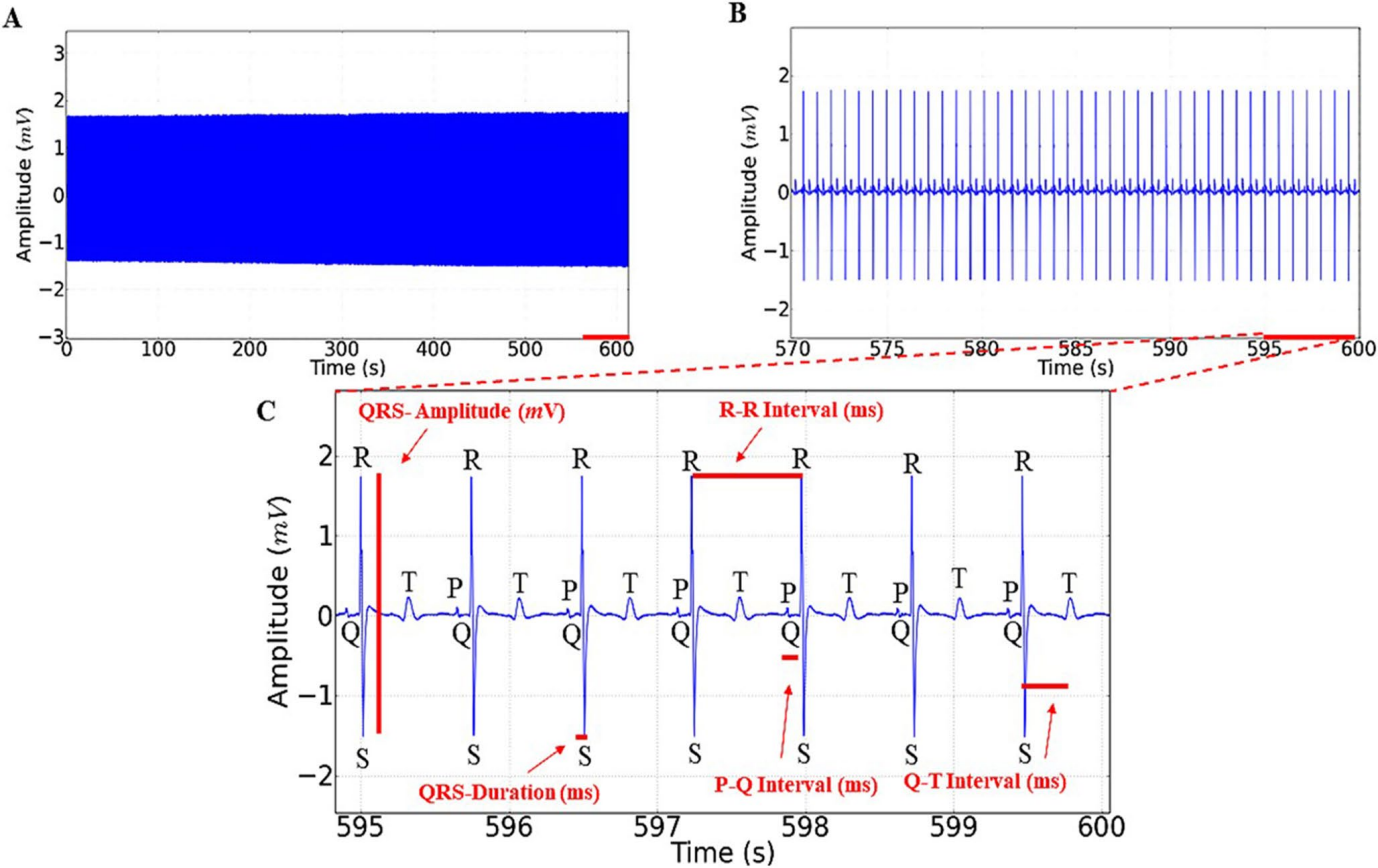
Electrocardiogram of the control group demonstrating cardiac activity of Colossoma macropomum (**A**). Amplification of the recording over 30 s (570–600 s) showing sinus rhythm (**B**). 5-second amplification (595–600 s) highlighting in red the morphological elements to be analyzed: Amplitude (mV), R-R Interval (s), P-Q Interval (s), Q-T Interval (s), and QRS Complex Duration (s) (**C**)

Figure 3A, B, and C analyze the electrical activity of the heart in *Colossoma macropomum* from the control group. The image presents three panels illustrating different aspects of the electrocardiogram (ECG) recorded over time. Cardiac activity in *Colossoma macropomum* in the control group shows a mean frequency of 86.67 ± 3.00 bpm and an amplitude of 1.99 ± 0.32 mV (Fig. 3A). In the 30-second amplification, sinus rhythm can be observed, with all cardiac deflections showing morphological elements: atrial activity represented by the P wave, ventricular activity represented by the QRS complex, and ventricular repolarization represented by the T wave (Figs. 3B and C).

**Fig. 4:**
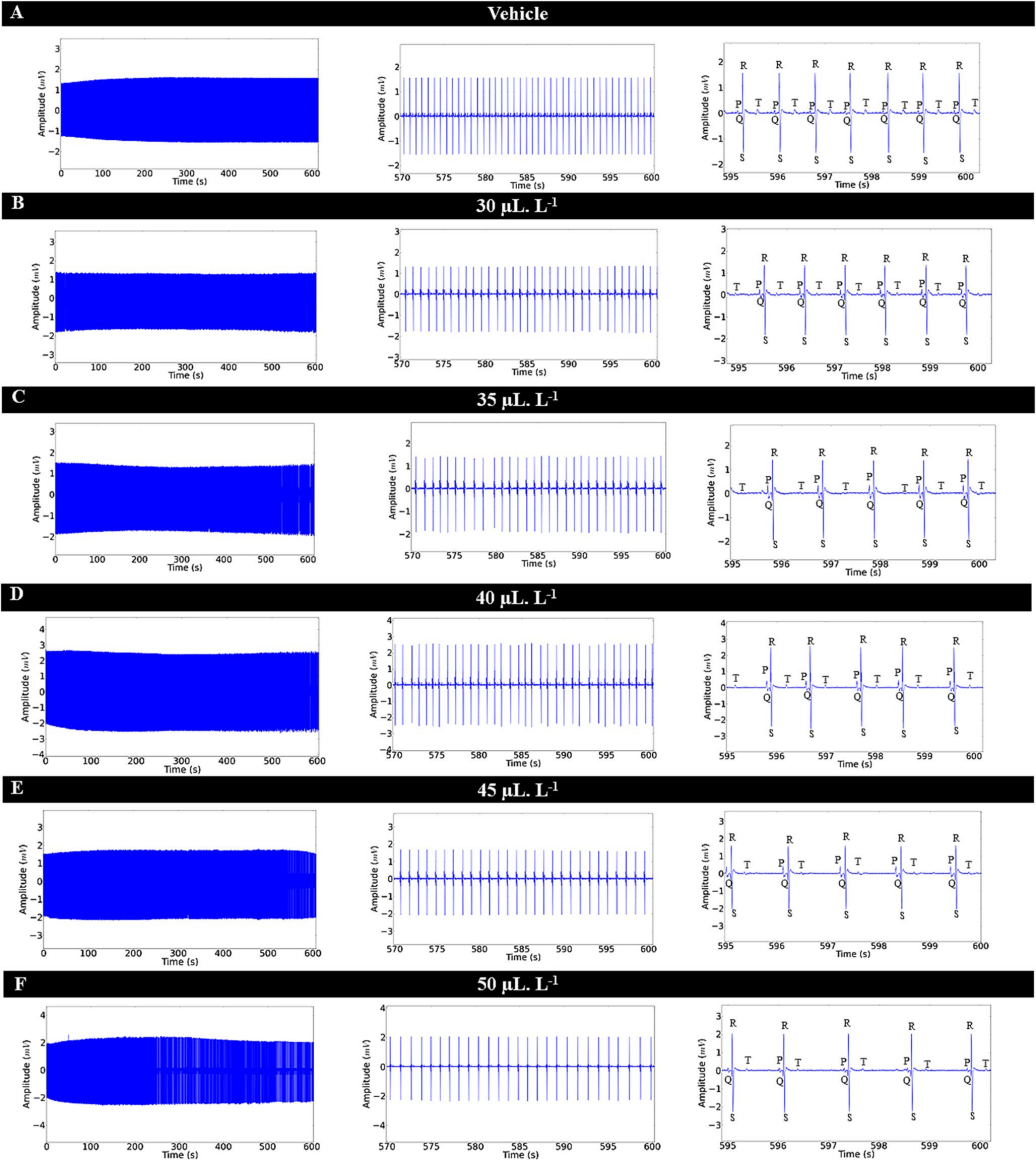
ECG of Colossoma macropomum during a 10-minute immersion bath with ENCO (left), amplification of the 30-second final segment (570–600 s) showing cardiac deflections (center), and amplification of the ECG of Colossoma macropomum during immersion bath with representation of morphological elements (right) for the following groups: vehicle (**A**); treated with 30 µL.L^− 1^ (**B**); treated with 35 µL.L^− 1^ (**C**); treated with 40 µL.L^− 1^ (**D**); 45 µL.L^− 1^ (E); and 50 µL.L^− 1^ (**F**)

The control group showed a heart rate of 86.67 ± 3.00 bpm, which was similar to the vehicle group (*p* = 0.9335). The group treated with 30 µL. L^− 1^ showed an 18.98% decrease in cardiac activity over 10 min of exposure. The groups treated with 35 µL. L^− 1^ (31.02%), 40 µL. L^− 1^ (31.27%), 45 µL. L^− 1^ (31.54%), and 50 µL. L^− 1^ (33.85%) exhibited respective decreases in cardiac activity (Figs. 4A, B, C, D, E, and F). The heart rhythm during the experiment with increasing concentrations of ENCO remained sinusoidal (Fig. 4). Bradycardia was found to be concentration-dependent; however, for the groups treated with 35 µL. L^− 1^, 40 µL. L^− 1^, and 45 µL. L^− 1^, the percentage of bradycardia was similar (Figs. 4C, D, and E).

**Fig. 5:**
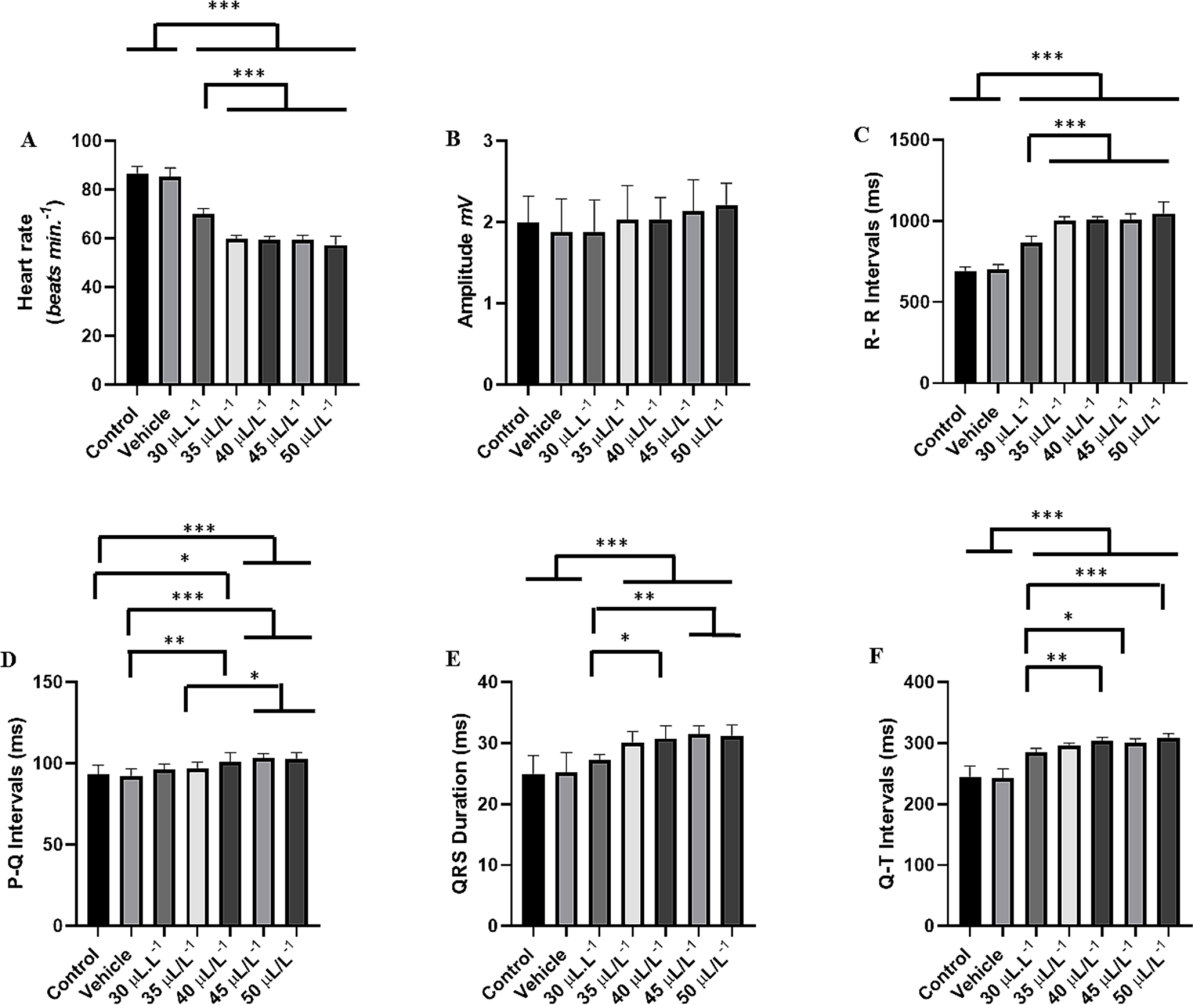
The graphs illustrate the mean values of heart rate (bpm) (**A**), QRS amplitude (mV) (**B**), R-R interval (ms) (**C**), P-Q interval (ms) (**D**), QRS duration (ms), and Q-T interval (ms) during recovery after exposure to ENCO at concentrations of 30 µL.L-1, 35 µL.L^− 1^, 40 µL. L^− 1^, 45 µL. L^− 1^, and 50 µL. L^− 1^. (ANOVA followed by Tukey; *p < 0.05; **p < 0.01; *p < 0.001; *n* = 9)

Heart rate was characterized by bradycardia. The control group had a mean of 86.67 ± 3.0 bpm, which was similar to the vehicle group (*p* = 0.9335) and higher than all other treated groups. The group treated with 30 µL. L^− 1^ had a mean of 70.22 ± 2.108 bpm, which was higher than the other treated groups. The group treated with 35 µL. L^− 1^ (59.78 ± 1.33 bpm) was similar to the groups treated with 40 µL. L^− 1^, 45 µL. L^− 1^, and 50 µL. L^− 1^ (*p* = 0.4468) (Fig. 5A).

The mean QRS amplitude for the control group was 1.99 ± 0.327 mV, similar to the other groups during anesthetic induction (F (6.56) = 1.026; *p* = 0.4183) (Fig. 5B).

The mean R-R interval for the control group was 692.4 ± 24.20 ms, similar to the vehicle group (*p* = 0.996), but higher than in the other groups. The group treated with 30 µL. L^− 1^ had a mean of 865.9 ± 42.61 ms, which was higher than in the other treated groups. The group treated with 35 µL. L^− 1^ (1003 ± 24.53 ms) was similar to the groups treated with 40 µL. L^− 1^, 45 µL. L^− 1^, and 50 µL. L^− 1^ (*p* = 0.2803) (Fig. 5C).

The mean P-Q interval for the control group was 93.56 ± 5.38 ms, similar to the vehicle group (*p* = 0.9920), and similar to 30 µL. L^− 1^ (*p* = 0.8258) and 35 µL. L^− 1^ (*p* = 0.6018), but shorter than in the other treated groups. The group treated with 30 µL. L^− 1^ (96.33 ± 3.42 ms) was similar to the group treated with 40 µL. L^− 1^ (*p* = 0.2788). The groups treated with 35 µL. L^− 1^, 40 µL. L^− 1^, 45 µL. L^− 1^, and 50 µL. L^− 1^ were similar (*p* = 0.0628) (Fig. 5D).

The mean duration of the QRS complex for the control group was 24.89 ± 3.060 ms, similar to the vehicle group and 30 µL. L^− 1^ (*p* = 0.3022). For the group treated with 30 µL. L^− 1^ (27.22 ± 0.971 ms), it was similar to the group treated with 35 µL. L^− 1^ (*p* = 0.133). The groups treated with 35 µL. L^− 1^ (30.00 ± 1.93 ms) were similar to the groups treated with 40 µL. L^− 1^, 45 µL. L^− 1^, and 50 µL. L^− 1^ (*p* = 0.8133) (Fig. 5E).

For the control group, the mean Q-T interval was 244.8 ± 18.22 ms, similar to the vehicle group (*p* = 0.999), but shorter than in the other groups. The group treated with 30 µL. L^− 1^ (285.4 ± 5.981 ms) was similar to the group treated with 35 µL. L^− 1^ (*p* = 0.3342). The group treated with 35 µL. L^− 1^ (296.0 ± 4.33 ms) was similar to the other treated groups (*p* = 0.1560) (Fig. 5F).

**Fig. 6:**
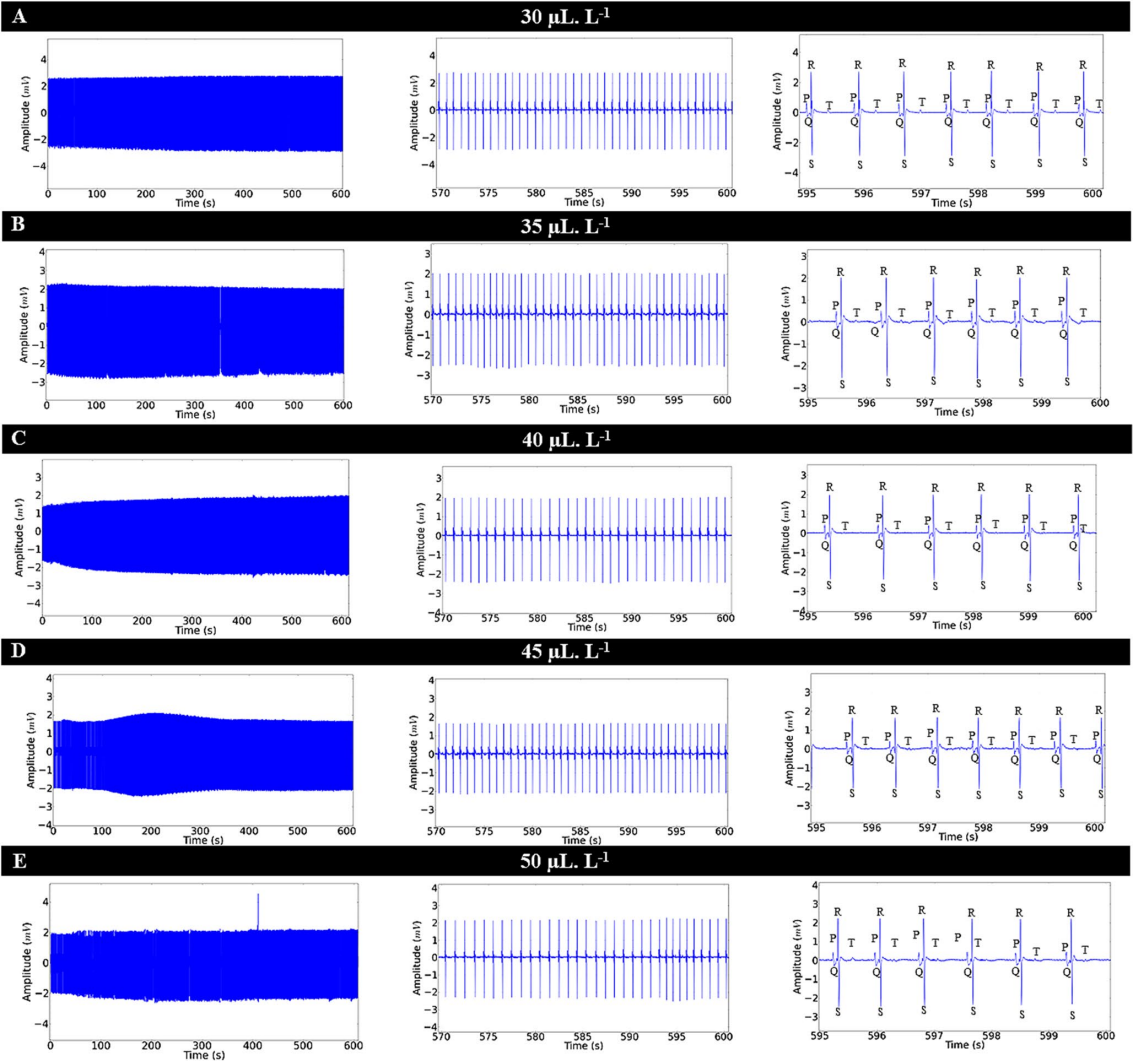
ECG of Colossoma macropomum during the recovery period after immersion treatment with ENCO (left), amplification of the 30-second final segment (570–600 s), showing characteristic ECG deflections with absence of arrhythmias (center), and amplification of cardiac activity of Colossoma macropomum during recovery after immersion for 5 s (595–600 s) (right) for treatments: 30 µL. L^-1^ (**A**); 35 µL. L^-1^ (**B**); 40 µL. L^-1^ (**C**); 45 µL. L^-1^ (**D**); and 50 µL. L^-1^ (**E**)

During the recovery period, reversibility of ECG alterations was observed (Figs. 6A, B, C, D, and E) after the removal of ENCO at concentrations of 30 µL. L^− 1^, 35 µL. L^− 1^, 40 µL. L^− 1^, 45 µL. L^− 1^, and 50 µL. L^− 1^. The heart rate for the control group was 86.67 ± 3.00 bpm. For the group treated with 30 µL. L^− 1^, the recovery was 93.33% relative to the control group. The other groups showed the following recovery percentages relative to the control: 35 µL. L^− 1^ (87.94%); 40 µL. L^− 1^ (84.60%); 45 µL. L^− 1^ (83.32%); and 50 µL. L^− 1^ (83.07%).

**Fig. 7:**
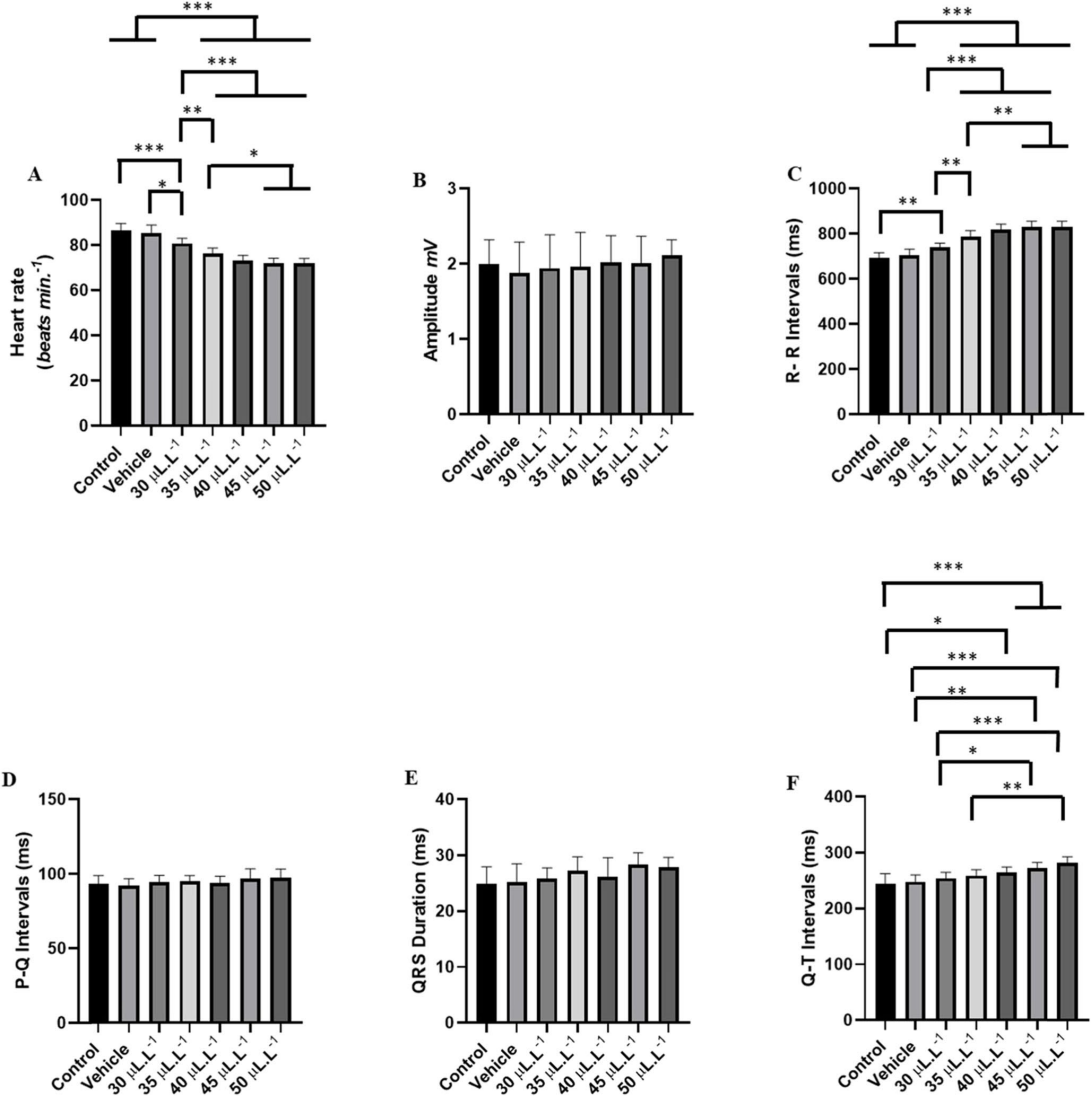
The graphs show the means of heart rate (bpm) (**A**), QRS amplitude (mV) (**B**), R-R interval (ms) (**C**), P-Q interval (ms) (**D**), QRS duration (ms), and Q-T interval (ms) during recovery after contact with ENCO at concentrations of 30µL. L^-1^, 35µL. L^-1^, 40µL. L^-1^, 45µL. L^-1^, and 50µL. L^-1^. (ANOVA followed by Tukey; *p < 0.05; **p < 0.01; ***p < 0.001; *n* = 9)

During the recovery period of heart rate after contact with ENCO, it was shown to be concentration-dependent, meaning that the higher the concentration, the longer the time required for the heart to revert to normal. The control group had a mean frequency of 86.67 ± 3.00 bpm, which was similar to the vehicle group (*p* = 0.9314) and higher than the other groups, indicating slow reversibility. The group treated with 30µL. L^− 1^ had a mean frequency of 80.89 ± 2.261 bpm, which was higher than that of the other treated groups. The group treated with 35µL. L^− 1^ (76.22 ± 2.53 bpm) was similar to the group treated with 40µL. L^− 1^ (*p* = 0.2437). For the groups treated with 40µL. L^− 1^ (73.33 ± 2.23 bpm), the frequency was similar to the groups treated with 45µL. L^− 1^ and 50µL. L^− 1^ (*p* = 0.9314) (Fig. 7A).

During recovery, the amplitudes of the controls showed a mean of 1.99 ± 0.327 mV, which was similar to the other recovery groups (F (6, 56) = 0.3281; *p* = 0.9194) (Fig. 7B).

The mean R-R intervals during recovery for the control group were 692.4 ± 24.20 ms, similar to the vehicle group (*p* = 0.9608), and lower than the other groups. The vehicle group (703.7 ± 28.79 ms) was similar to the group treated with 30µL. L^− 1^ (*p* = 0.0569). The group treated with 35µL. L^− 1^ (787.7 ± 27.06 ms) was similar to the group treated with 40µL. L^− 1^ (*p* = 0.143). The group treated with 40µL. L^− 1^ (818.2 ± 24.87 ms) was similar to the groups treated with 45µL. L^− 1^ and 50µL. L^− 1^ (*p* = 0.9283) (Fig. 7C).

The mean P-Q intervals during recovery for the control group were 93.56 ± 5.388 ms, similar to the other recovery groups (F (6, 56) = 1.22; *p* = 0.309) (Fig. 7D). The duration of the QRS complex during recovery showed a mean for the control group of 24.89 ± 3.06 ms, which was similar to the other groups (F (6, 56) = 2.23; *p* = 0.0532) (Fig. 7E).

During recovery, the Q-T interval for the control group had a mean of 244.8 ± 18.22 ms, which was similar to the vehicle group, as well as the groups treated with 30µL. L^− 1^ and 35µL. L^− 1^ (*p* = 0.1772). The vehicle group was similar to the group treated with 40µL. L^− 1^ (*p* = 0.071). The group treated with 30µL. L^− 1^ (254.6 ± 10.62 ms) was similar to the groups treated with 40µL. L^− 1^ and 45µL. L^− 1^ (*p* = 0.5532). The group treated with 35µL. L^− 1^ (259.0 ± 10.81 ms) was similar to the groups treated with 40µL. L^− 1^ and 45µL. L^− 1^ (*p* = 0.1916). The mean Q-interval for the group treated with 40µL. L^− 1^ (264.8 ± 9.78 ms) was similar to the groups treated with 45µL. L^− 1^ and 50µL. L^− 1^ (*p* = 0.0586) (Fig. 7F).

### Underlying mechanisms of eugenol action demonstrate the allosteric participation of the GABAA receptor, enabling the identification of synergism between eugenol and N. Cataria essential oil

**Fig. 8:**
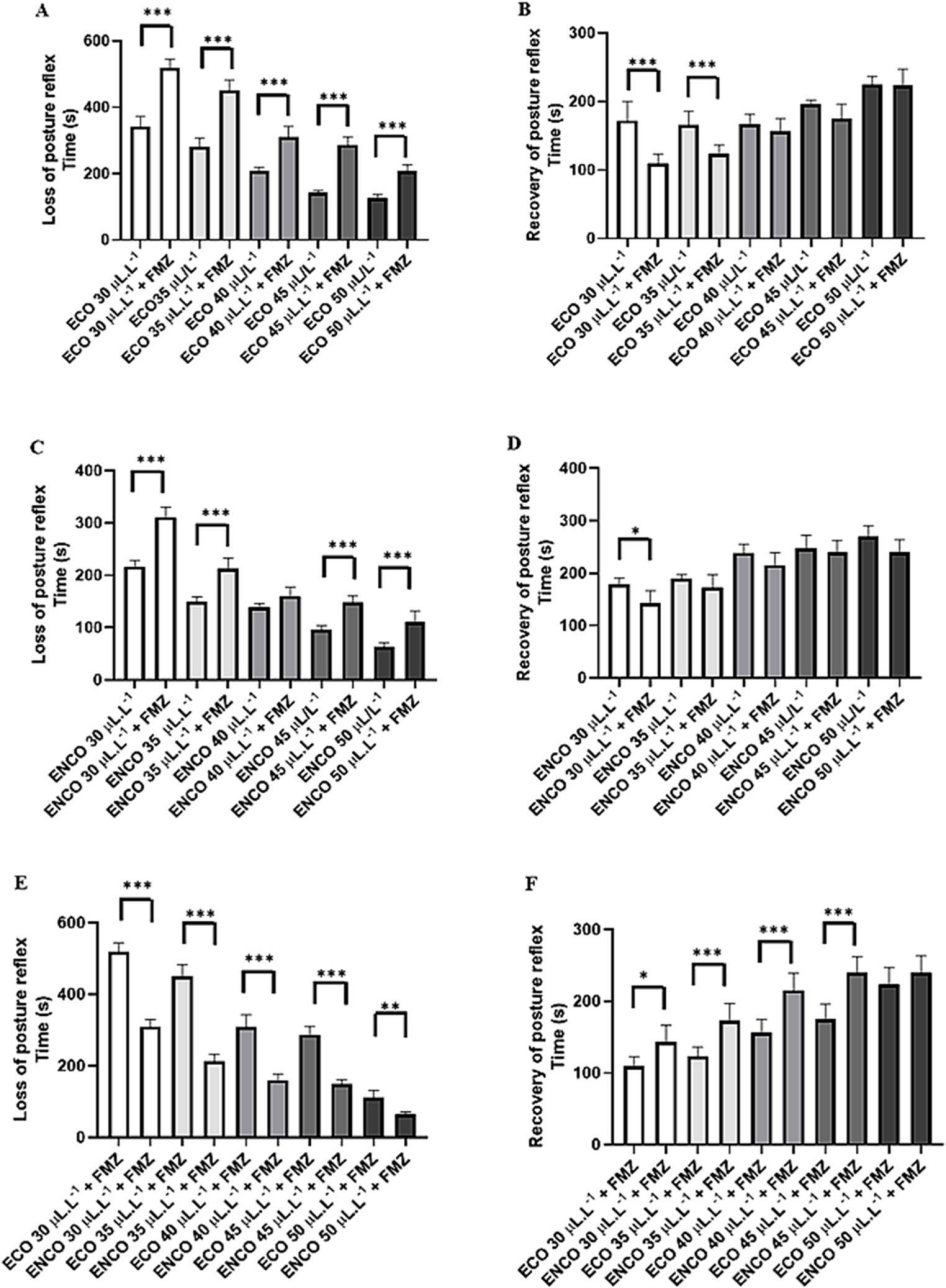
Graphs demonstrating induction and recovery during immersion bath with ECO and ENCO without prior treatment with flumazenil and 15 min after treatment with flumazenil 1 mg/kg i.p. For evaluation of the following groups: (**a**) ECO Induction; (**b**) ECO recovery; (**c**) ENCO induction; (**d**) ENCO recovery; (**e**) ECO and ENCO induction and (**f**) ECO and ENCO recovery

During the test with flumazenil 1 mg/kg i.p. 15 min before treatment with ECO, the animals presented greater latency for loss of the posture reflex with a 51.63% increase in latency for loss of the posture reflex for the group treated with ECO 30 µL. L^− 1^. The latency increased considerably for the other groups 35 µL. L^− 1^ (60.71%), 40 µL. L^− 1^ (49.18%), 45 µL. L^− 1^ (100.48%) and 50 µL. L^− 1^ (65.08%) (Fig. 8A).

Flimazenil decreased the latency for recovery of the posture reflex after ECO treatment for the groups treated with 30 µL. L^− 1^, 35 µL. L^− 1^, however, for the groups treated with 40 µL. L^− 1^ (*p* = 0.9636), 45 µL. L^− 1^ (*p* = 0.3034) and 50 µL. L^− 1^ (*p* = 0.999) the latencies were similar (Fig. 8B).

For the groups treated with ENCO, pretreatment application of flumazenil increased the latency to induction in the groups treated with 30 µL. L^− 1^ (43.49%), 35 µL. L^− 1^ (42.95%), 45 µL. L^− 1^ (55.10%) and 50 µL. L^− 1^ (73.56%). However, the group treated with 40 µL. L^− 1^ was similar in the groups treated and not treated with flumazenil (*p* = 0.058) (Fig. 8C).

During recovery, the groups in the ENCO group, the group treated with flumazenil and ENCO at 30 µL. L^− 1^ presented lower latency. The groups tested with 35 µL. L^− 1^ (*p* = 0.7554), 40 µL. L^− 1^ (*p* = 0.3437), 45 µL. L^− 1^ (*p* = 0.9969) and 50 µL. L-1 (*p* = 0.0993) were similar to the groups treated with flumazenil (Fig. 8D).

Comparing the ECO and ENCO groups that previously received treatment with flumazenil, it can be observed that the ECO group presented a greater latency for loss of the posture reflex when compared to ENCO. Thus, the groups treated with ECO had latencies for anesthetic induction. The group that received 30 µL. L^− 1^ had a 66.66% greater latency. The groups 35 µL. L^− 1^ (111.26%), 40 µL. L^− 1^ (94.30%), 45 µL. L^− 1^ (92.94%) and 50 µL. L^− 1^ (73.56%) (Fig. 8E).

The latency to recovery was lower for the ECO group treated with flumazenil than for the groups treated with 30 µL. L^− 1^, 35 µL. L^− 1^, 40 µL. L^− 1^ and 45 µL. L^− 1^. However, the groups treated with 50 µL. L^− 1^ had similar latencies (*p* = 0.8152) (Fig. 8F).

## Discussion

Initially, it is important to highlight that the proportional combination of *Nepeta cataria* oil and eugenol to evaluate the synergistic effect by potentiation during the induction and recovery phases of anesthesia in fish has not yet been investigated, representing an innovative aspect of the present study. Numerous studies have investigated the effects of eugenol as a standard anesthetic in different fish species, including *Colossoma macropomum*. For example, Roubach et al. (2005) identified that the required dose of eugenol to achieve an anesthetic response in juvenile and subadult tambaquis is 65 mg.L⁻¹, and exposure for more than 30 min did not result in mortality (Roubach et al. 2005). Furthermore, previous studies have demonstrated that *Nepeta cataria* essential oil produces significant anesthetic effects, reinforcing its application in this species (dos Santos et al. 2024; de Souza et al. 2019). Thus, both anesthetics are investigated in anesthetic procedures for tambaqui, highlighting the potential synergistic effect between these substances.

Our experiments demonstrated that the combination of eugenol with *Nepeta cataria* essential oil in equivalent proportions increases the efficacy of anesthetic induction, reducing the latency time. The reduction in induction time was significantly greater at higher concentrations of the eugenol and *Nepeta cataria* combination. Sousa et al. (2021) reported an induction time of 100 s using a concentration of 100 mg.L⁻¹ of eugenol in tambaqui (Sousa et al. 2021). Compared to the present study, the higher concentrations of eugenol and *Nepeta cataria* were able to induce anesthesia in a shorter time than reported in the previous study. Regarding anesthetic recovery time, the present study showed a recovery time similar to that reported by (Sousa et al. 2021). The anesthetic induction and recovery times remained within the period suggested by the literature for safe anesthesia in fish (Park et al. 2008; Ross et al. 2009).

Studies on the synergy between substances for use in anesthetic procedures in fish are essential, as there are reports in the literature indicating that a single substance, when used alone, may not achieve the desired effect. However, when combined with another substance, a more effective and satisfactory response is observed. The study by Nuanmanee et al. (2024) revealed that at concentrations of 100 and 200 mg.L⁻¹, the compound 1,8-cineol was not able to induce anesthesia in guppies. However, when combined with eugenol, the animals exhibited a more efficient anesthetic response with a shorter induction time. Similarly, Tchobanov et al. (2024) reported the synergistic effect between lidocaine, clove oil, and 2-phenoxyethanol. These studies support our findings, which demonstrate the synergistic effect through anesthetic potentiation in tambaqui, highlighting the importance of further investigations in this area.

Moreover, this combination makes anesthetic procedures safer by minimizing side effects, such as deep cardiovascular depression caused by excessive use of eugenol. The results presented in this study show that exposure to the essential oil of Nepeta cataria and eugenol (ENCO) induced bradycardia with sinus rhythm, without the presence of indicators of cardiac arrhythmias in *Colossoma macropomum*. The observed bradycardia was dose-dependent, with a reduction in heart rate of up to 33.85% in fish exposed to the highest concentration of ENCO (50 µL. L⁻¹). This effect is consistent with studies indicating that eugenol may act as a modulator of ion channels, affecting electrical conduction in the myocardium and, consequently, reducing heart rate, blood pressure, and local anesthetic action capable of blocking nerve bundles (Le Daré et al. 2022; Hwang et al. 2020; Interaminense et al. 2007; Lahlou et al. 2004; Park et al. 2009). The bradycardia presented by the use of ENCO was easily reversible, which demonstrates its safety, since this clinical sign, depending on its intensity, can impair the animal’s hemodynamics, hinder tissue oxygenation, and cause prolonged return due to compromised perfusion.

In addition, a clear loss of muscle tone was observed during anesthetic induction, followed by the recovery of tone after treatment, confirming the synergistic interaction between the essential oils tested. The time for muscle tone loss was significantly shorter at concentrations of 40–50 µL. L⁻¹ of ENCO. The study by Souza et al. (2019) presented similar results, reporting muscle tone loss within the same time range when using a concentration of 175 µL. L⁻¹ of *Nepeta cataria* essential oil in tambaqui. On the other hand, Barbas et al. (2021) observed a faster loss of muscle tone with the use of eugenol at a concentration of 65 µL. L⁻¹ (Barbas et al. 2021). However, both of the previous studies reported longer periods for muscle tone recovery, especially with the use of eugenol. Thus, the present work supports the synergistic effect on muscle tone loss and recovery between the substances tested.

To demonstrate the synergistic effect between eugenol and *Nepeta cataria* essential oil, flumazenil, a GABA-A benzodiazepine receptor antagonist, was administered 15 min prior to treatment with ECO or ENCO during the anesthetic induction and recovery processes. In the groups treated with ECO or ENCO, an increase in the latency time for induction was observed, suggesting that flumazenil interferes with the effect of eugenol. These results indicate that eugenol exerts its anesthetic action through an allosteric interaction with the GABA-A receptor. As reported by (Delgado-Marín et al. 2017; Ding et al. 2014; Yano et al. 2006), eugenol exhibits multiple biological effects, including anticonvulsant, anesthetic, and analgesic activities, mediated by the activation of ionotropic γ-aminobutyric acid (GABA) receptors. When comparing the ECO and ENCO groups previously treated with flumazenil, it was observed that the ECO group exhibited a longer latency for anesthetic induction and a shorter latency for recovery, highlighting the influence of flumazenil on these processes.

Furthermore, despite the occupation of GABA-A receptors by flumazenil, the anesthetic induction time with ENCO was shorter. This can be attributed to the different mechanisms of action of the compounds studied. Aydin et al. (1998) suggest a possible interaction of nepetalactone, the major compound in *Nepeta cataria* essential oil, with opioid receptors (Aydin et al. 1998). In fish, µ and κ opioid receptors are present in the brain (Hadfield and Clayton 2021; Rodrigues et al. 2019; Alho da Costa et al. 2026), indicating a potential interaction of this compound with these receptors, which could promote a more pronounced anesthetic response. Additionally, the study demonstrated the stability of the interaction between eugenol and the Oprk1 protein, activating the opioid pathway, inhibiting neurotransmitter release, and exerting sedative and analgesic effects (Zeng et al. 2024). Thus, the different compounds may act through distinct pathways, making their combined administration feasible for enhancing the anesthetic effect. At lower concentrations of ENCO, flumazenil showed a greater capacity to restore the postural reflex, indicating pharmacological competition for the site of action. However, at higher concentrations, the recovery time for ENCO was similar to that of flumazenil. This may indicate that not all of the effect is related to the GABA receptor, with other underlying pathways contributing to its effect, involving the complexity of the organism.

The limitations of our study are related to its performance only on young animals, the absence of hematological analyses that indicate stress, such as cortisol, blood glucose, and oxidative stress meters, which complement the safety of using the anesthetic combination, and the lack of analytical confirmation (GC-MS) of the indications made by the oil sample manufacturer, as well as the LC50 for the oils used.

## Conclusion

This study demonstrated that the components of Nepeta cataria essential oil and eugenol exhibit synergistic action and can be used in doses of 30 to 50 µL.L^− 1^ in the tested species, observing good muscle relaxation and favorable electrocardiographic parameters, with safe pharmacological reversibility. The synergistic effect allowed the use of low concentrations to achieve deep anesthetic planes with minimal alterations in cardiac function, in addition to rapid induction and safe and gradual recovery. Furthermore, we demonstrated the allosteric effect of eugenol in potentiating the GABA-A receptor, one of the pathways that contribute to the maintenance of central nervous system depression in animals.

## Supplementary Information

Below is the link to the electronic supplementary material.

Supplementary Material 1

Supplementary Material 2

## Data Availability

No datasets were generated or analysed during the current study.
