# Supplementary material for: Association of Nepeta cataria L. essential oil and eugenol: synergism and safe anesthesia in tambaqui Colossoma macropomum (Cuvier, 1818)
Source: Vet Res Commun. 2026 May 28;50(4):353. doi: 10.1007/s11259-026-11298-x (PMC13219177; doi:10.1007/s11259-026-11298-x)
Supplement: Supplementary file 1 — Supplementary Material 1 [file 11259_2026_11298_MOESM1_ESM.docx]

| 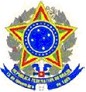 | **Ministry of Education - Brazil**  **Federal University of Pará**  Belém – PA- Brazil | 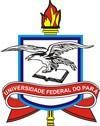 |
| --- | --- | --- |

# Through the study entitled "*Association of Nepeta cataria L. essential oil and eugenol: synergism and safe anesthesia in Colossoma macropomum (Cuvier, 1818"* we can consider the following Highlights:

- The combination of Nepeta cataria essential oil and eugenol showed clear synergism.
- The association reduced induction latency and preserved safe anesthetic recovery.
- Electromyographic data confirmed reversible muscle relaxation at all concentrations.
- Electrocardiographic recordings indicated mild, reversible bradycardia during exposure.
- Flumazenil confirmed eugenol’s GABA<sub>A</sub>-mediated action and complementary mechanisms.
