# Supplementary figures and images for: Association of Nepeta cataria L. essential oil and eugenol: synergism and safe anesthesia in tambaqui Colossoma macropomum (Cuvier, 1818)
Source: Vet Res Commun. 2026 May 28;50(4):353. doi: 10.1007/s11259-026-11298-x (PMC13219177; doi:10.1007/s11259-026-11298-x)

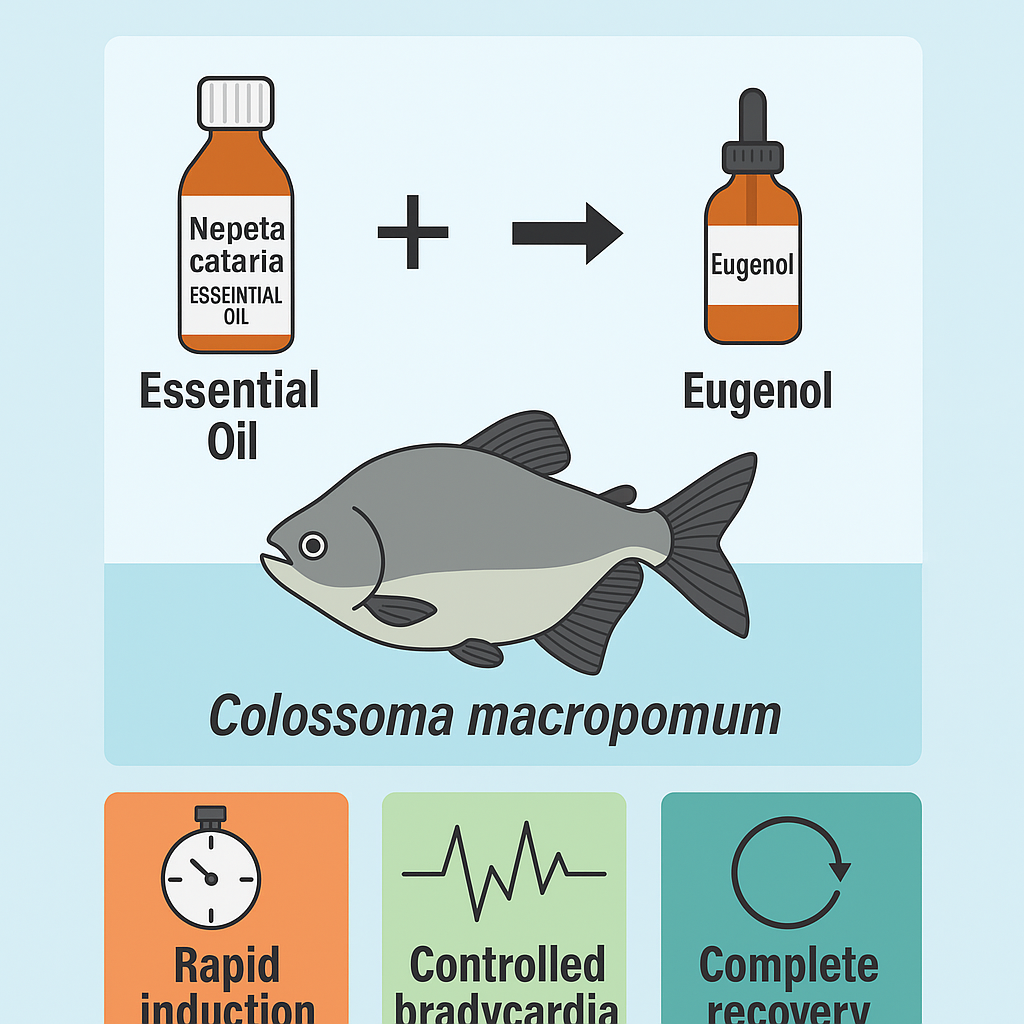

Supplement: Supplementary file 2 — Supplementary Material 2 [file 11259_2026_11298_MOESM2_ESM.png]
